# Rare intronic variants of TCF7L2 arising by selective sweeps in an indigenous population from Mexico

**DOI:** 10.1186/s12863-016-0372-7

**Published:** 2016-05-26

**Authors:** Jose Luis Acosta, Alma Cristal Hernández-Mondragón, Laura Carolina Correa-Acosta, Sandra Nathaly Cazañas-Padilla, Berenice Chávez-Florencio, Elvia Yamilet Ramírez-Vega, Tulia Monge-Cázares, Carlos A. Aguilar-Salinas, Teresa Tusié-Luna, Laura del Bosque-Plata

**Affiliations:** Laboratorio de Nutrigenética y Nutrigenómica, Instituto Nacional de Medicina Genómica, Periférico Sur No. 4809, Col. Arenal Tepepan, 14610 Mexico City, Mexico; Instituto de Investigaciones Biomédicas, UNAM, Unidad de Biología Molecular y Medicina Genómica, UNAM/INCMNSZ, 04510 Mexico City, Mexico.; Departamento de Microbiología y Patología, Centro Universitario de Ciencias de la Salud, Universidad de Guadalajara, Sierra Mojada No. 950, Puerta 7, Edificio O, Planta Baja, Col. Independencia, 44340 Guadalajara, Jalisco Mexico; Departamento de Endocrinología y Metabolismo, Instituto Nacional de Ciencias Médicas y Nutrición, Vasco de Quiroga 15, 14000 Mexico City, Mexico; Instituto Politécnico Nacional, Centro Interdisciplinario de Investigación para el Desarrollo Integral Regional (CIIDIR)-Unidad, Blvd, Juan de Dios Bátiz Paredes #250, 81101 Sinaloa, Mexico

**Keywords:** TCF7L2 gene, Type 2 diabetes, Genetic association, Sweeps selection, Recombination hotspots

## Abstract

**Background:**

Genetic variations of the TCF7L2 gene are associated with the development of Type 2 diabetes (T2D). The associated mutations have demonstrated an adaptive role in some human populations, but no studies have determined the impact of evolutionary forces on genetic diversity in indigenous populations from Mexico. Here, we sequenced and analyzed the variation of the TCF7L2 gene in three Amerindian populations and compared the results with whole-exon-sequencing of Mestizo populations from Sigma and the 1000 Genomes Project to assess the roles of selection and recombination in diversity.

**Results:**

The diversity in the indigenous populations was biased to intronic regions. Most of the variation was low frequency. Only mutations rs77961654 and rs61724286 were located on exon 15. We did not observe variation in intronic region 4–6 in any of the three indigenous populations. In addition, we identified peaks of selective sweeps in the mestizo samples from the Sigma Project within this region. By replicating the analysis of association with T2D between case-controls from the Sigma Project, we determined that T2D was most highly associated with the rs7903146 risk allele and to a lesser extent with the other six variants. All associated markers were located in intronic region 4–6, and their r^2^ values of linkage disequilibrium were significantly higher in the Mexican population than in Africans from the 1000 Genomes Project. We observed reticulations in both the haplotypes network analysis from seven marker associates and the neighborNet tree based on 6061 markers in the TCF7L2 gene identified from all samples of the 1000 Genomes Project. Finally, we identified two recombination hotspots in the upstream region and 3’ end of the TCF7L2 gene.

**Conclusions:**

The lack of diversity in intronic region 4–6 in Indigenous populations could be an effect of selective sweeps generated by the selection of neighboring rare variants at T2D-associated mutations. The survivors’ variants make the intronic region 4–6 the area of the greatest population differentiation within the TCF7L2 gene. The abundance of selective peak sweeps in the downstream region of the TCF7L2 gene suggests that the TCF7L2 gene is part of a region that is in constant recombination between populations.

**Electronic supplementary material:**

The online version of this article (doi:10.1186/s12863-016-0372-7) contains supplementary material, which is available to authorized users.

## Background

Type 2 diabetes (T2D) is a leading global health problem. The diabetic population is estimated to increase from 366 million to 552 million by the year 2030. T2D-related genetic variants explain only a small portion of the heritability of T2D. Ethnic differences in the prevalence of T2D may be attributable to distinct genetic backgrounds of these populations. High-throughput sequencing and genotype data for ethnic groups other than those in which most of the known variants have been discovered will enable the discovery of new variants and an exploration of their role in T2D [1]. Genetic variants of TCF7L2 have been associated with T2D in nearly all ethnic groups studied [2–7]. TCF7L2 is a crucial transcription factor of the Wnt signaling pathway; this pathway plays a role in development and comprises a complex network of protein interactions that regulate cellular intercommunication at multiple levels [8]. TCF7L2 is most strongly associated with T2D in populations of Caucasian origin [2]. Subsequent whole gene analyses of different populations, such as Chinese [9, 10] and Mexican American populations [11], have revealed other variants associated with T2D. In addition, polymorphisms in the TCF7L2 gene that are associated with T2D exhibit patterns of variation and divergence in concordance with natural selection in Caucasian populations [12], but the relationship of this pattern to metabolic characteristics is not completely understood [13]. The effect of natural selection on this gene in a Latin population is not known. Therefore, in this study, we investigated new variants in the TCF7L2 gene in a Mexican population and analyzed the selective sweep patterns of the TCF7L2 gene polymorphisms using exome-sequencing data from the Sigma Project and 1000 Genomes Project.

## Results

### Mutations in the Mexican population

TCF7L2 is a transcription factor in the Wnt signaling pathway and is expressed in many tissues, including fat, liver and pancreatic islets of Langerhans [14]. Genetic variations of the TCF7L2 gene, either in introns or exons, have been associated with T2D, and these markers have been observed in several individuals from different populations, including the Mexican population [2, 15, 16]. Risk alleles associated with gestational diabetes have also been identified in the Mexican population, but the degree of Amerindian ancestry in the subjects of these studies was unknown [17]. Therefore, to estimate the variability of the TCF7L2 gene in a Mexican population with high Amerindian ancestry, we sequenced three groups of indigenous people and one group of mixed race from Mexico (See Methods) comprising 60 Mazatecan, 34 Nahua, 30 Zapotecan and 30 Mestizos.

Although the primers were designed primarily for exons of the TCF7L2 gene (See Methods), most of the variation identified in the Mexican population occurred in introns, and only six variants were shared with the 1000 Genomes Project and the Sigma Project (Table 1). We scanned the diversity of the TCF7L2 gene in 4,060 exomes (see Methods) and identified only 80 exonic variants, corresponding to a nucleotide diversity of 0.00147 (data not shown). The low diversity is also reflected in the allelic frequency of the rs77961654 (C/A) missense mutation in exon 15, which was observed in the Indigenous (Mexican) population, the 1000 Genomes Project, and the Sigma Project (both cases and controls). The minor allelic frequency (MAF) of rs77961654 (C/A) was lower in the Sigma Project (0.0896552) than in the 1000 Genomes Project (0.129793). Similarly, the MAF of the rs61724286 (C/G) polymorphism in exon 15 of the TCF7L2 gene in the Indigenous population (Table 1) was lower in the Sigma Project (0.000985222). The low MAF of the variants in exon 15 could suggest a purge of diversity in this region; however, the intronic variants identified in the Indigenous populations, which were also observed in the 1000 Genomes Project, exhibited a heterogeneous MAF. In particular, variants rs176632 (T/C) and var_10_114919595 (C/T) in intron 9 exhibited MAFs of 0.992412 (high) and 0.002396 (low), respectively. This heterogeneity in MAF (0.1984 and 0.000399) was also observed in the variants rs56913138 (G/T) and rs188695269 (G/A) in intron 6 (Table 1) and could be a footprint of selective processes of ancestral variants.

**Table 1:** Mutations identified in the Indigenous population and shared with the Sigma and 1000 Genome Projects. * Frequencies sorted by cases: controls; cases are defined as patients with type 2 diabetes

1000 Genomes Project		Sigma Project		Indigenous people								
MAF	Genotypes	MAF	Genotypes^a^	Variant Exonic/Intronic	Gene	Gene coordenate	dbSNP138	Chr	Start	End	Ref	Obs
0.1984	2007(G/G)/451(G/T)/46(T/T)			Intronic 6	Gene	191193	rs56913138	Chr10	114901201	114901201	G	T
0.000399	2503(G/G)/1(G/T)/0(T/T)			Intronic 6	Gene	193976	rs188695269	Chr10	114903984	114903984	G	A
0.992412	19(T/T)/303(T/C)/2182(C/C)			Intronic 9	Gene	201071	rs176632	Chr10	114911079	114911079	T	C
0.002396	2498(C/C)/6(C/G)/0(G/G)			Intronic 9	Gene	209587		Chr10	114919595	114919595	C	T
0.129793	2179(C/C)/301(C/A)/24(A/A)	0.0896552	C/C = 1564:1618 C/A = 331:320 A/A = 10:16	Exonic 15	Gene	215361	rs77961654	Chr10	114925369	114925369	C	A
		0.000985222	C/C = 1901:1950 C/G = 4:4	Exonic 15	Gene	215363	rs61724286	Chr10	114925371	114925371	C	G

^a^Genotypes

Frequencies sorted cases: controls, the cases defined by patients with type 2 diabetes

The majority of mutations identified in the Indigenous population were new and were located in noncoding regions (Table 2), particularly in introns 4, 6, 7, 11, 13, and 15, it was not possible to calculate the MAF based on the 1000 Genomes Project or Sigma Project. Due to the low number of mutations observed in the Indigenous population, these variations can be referred to as rare genetic variants. It has been hypothesized that mutations with a MAF of 0.5 – 1 % (lower frequency) and rare variations explain a substantial fraction of the heritability of common, complex diseases [18]. In the exome-sequencing, the excess of rare variations was consistent with explosive human population growth [19], and most of the mutations with functional impact were maintained by weak purification [20]. We assessed the functional impact of two exonic variants (rs77961654 and rs61724286) in the Indigenous population (see Methods) and did not find a deleterious impact of the TCF7L2 gene in either case. The lack of deleterious mutations (modify the function of the protein) suggests some type of selection inside of the gene.

**Table 2:** Novel mutations identified in the Indigenous population

Indigenous population								
Variant Exonic/Intronic	Gene	Gene coordenate	dbSNP138	Chr	Start	End	Ref	Obs
Intronic 4	TCF7L2	14402		Chr10	114724410	114724410	T	A
Intronic 4	TCF7L2	14404		Chr10	114724412	114724412	G	C
Intronic 6	TCF7L2	191411		Chr10	114901419	114901419	A	T
Intronic 6	TCF7L2	191412		Chr10	114901420	114901420	T	G
Intronic 6	TCF7L2	193490		Chr10	114903498	114903498	A	C
Intronic 6	TCF7L2	193491		Chr10	114903499	114903499	C	A
Intronic 7	TCF7L2	193908		Chr10	114903916	114903916	C	A
Intronic 7	TCF7L2	193913		Chr10	114903921	114903921	T	A
Intronic 7	TCF7L2	193940		Chr10	114903948	114903948	G	C
Intronic 7	TCF7L2	193951		Chr10	114903959	114903959	G	T
Intronic 7	TCF7L2	193967		Chr10	114903975	114903975	G	A
Intronic 7	TCF7L2	193975		Chr10	114903983	114903983	G	T
Intronic 7	TCF7L2	193982		Chr10	114903990	114903990	C	A
Intronic 7	TCF7L2	193987		Chr10	114903995	114903995	G	C
Intronic 7	TCF7L2	193988		Chr10	114903996	114903996	C	A
Intronic 7	TCF7L2	193995		Chr10	114904003	114904003	G	C
Intronic 7	TCF7L2	194007		Chr10	114904015	114904015	T	A
Intronic 7	TCF7L2	194012		Chr10	114904020	114904020	G	T
Intronic 7	TCF7L2	195524		Chr10	114905532	114905532	C	G
Intronic 11	TCF7L2	207481		Chr10	114917489	114917489	G	C
Intronic 11	TCF7L2	207483		Chr10	114917491	114917491	T	A
Intronic 11	TCF7L2	207485		Chr10	114917493	114917493	T	C
Intronic 11	TCF7L2	207486		Chr10	114917494	114917494	G	A
Intronic 11	TCF7L2	207487		Chr10	114917495	114917495	G	A
Intronic 11	TCF7L2	207488		Chr10	114917496	114917496	G	C
Intronic 11	TCF7L2	207492		Chr10	114917500	114917500	G	T
Intronic 11	TCF7L2	207495		Chr10	114917503	114917503	T	G
Intronic 11	TCF7L2	207497		Chr10	114917505	114917505	G	C
Intronic 11	TCF7L2	207499		Chr10	114917507	114917507	C	A
Intronic 11	TCF7L2	207500		Chr10	114917508	114917508	A	T
Intronic 11	TCF7L2	207501		Chr10	114917509	114917509	A	G
Intronic 11	TCF7L2	207502		Chr10	114917510	114917510	T	A
Intronic 11	TCF7L2	207504		Chr10	114917512	114917512	G	A
Intronic 13	TCF7L2	209536		Chr10	114919544	114919544	A	T
Intronic 13	TCF7L2	209555		Chr10	114919563	114919563	T	A
Intronic 13	TCF7L2	209562		Chr10	114919570	114919570	C	T
Intronic 13	TCF7L2	209571		Chr10	114919579	114919579	A	G
Intronic 15	TCF7L2	215224		Chr10	114925232	114925232	A	T
Intronic 15	TCF7L2	215229		Chr10	114925237	114925237	G	T
Intronic 15	TCF7L2	215230		Chr10	114925238	114925238	G	C

### Selection and recombination

To observe selection effects, we analyzed the sweep patterns in a region that included 1 megabase around the TCF7L2 gene using data from the Sigma Project (see Methods). The selective sweeps predict the pattern of excessive linkage disequilibrium (LD) in each of the two regions that flank a recently fixed beneficial mutation by strong positive selection [21]. The Cross Population Extend Haplotype Homozygosity Test (XPEHH) is highly powerful in detecting those with approximately fixed or fixed selected loci [22] and given the association of LD patterns and selective sweeps, we calculated statistics r^2^ and XP-EHH (see Methods) and we found that the values significant of r^2^ (higher > 0,6) in Latino population, flanked to regions with a strong positive selection (values significant of XP-EHH). It is worth mentioning that most of selected regions only in the Latino population (values positive of XP-EHH) were localized in regions adjacent to TCF7L2 gene. The upstream region included the genes MIR4295 and VTI1A, while the downstream region harbored the genes DCLRE1A and NHLRC2. This downstream region was located within of a block hard selection sweeps, which covers several genes involved in metabolism, for example, genes PLEKHS1 and MIR4483. However, the values significant of r^2^ were found in a region of soft selection sweeps in TCF7L2 gene (Additional files 1, 2 and 3) . In a context of disease association, the r^2^ statistic is often used in calculations of power to detect disease-susceptibility loci [23].

**Fig. 1:**
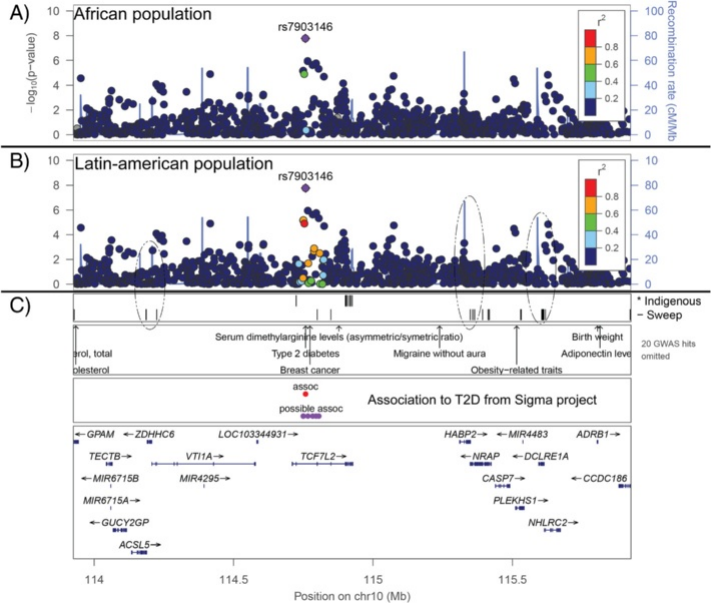
Dynamics of selective sweeps, recombination rate and linkage disequilibrium (LD) in the chr10:113925369–115925369 region (**a**) and (**b**), The y axis on the left side is the negative logarithm of the p-value of association of case-controls of the Sigma Project samples. The y axis on right side is the recombination rate (cM/Mb) calculated by the 1000 Genomes Project. The recombination peaks are represented by the solid blue line. r^2^ was 0–1 and was calculated for the African population only. The color spectrum of the spheres is a function of the r^2^ value. (**c**) The first segment is the genomic coordinates of chromosome 10 based on mutations identified in the Indigenous population and the selective sweep peaks determined only from the Sigma Project samples. The dashed black circles indicate overlap of the recombination hotspots with the selective sweep peaks. The second segment represent the location in chromosome 10 of the hits of the Sigma Project mutations against the GWAS databases. The third segment includes mutations associated with T2D. Red indicates associated mutations, and blue indicates mutations inside the limit cutoff of the Manhattan plot. The fourth segment is the genomic context of the analyzed region, with the exons and introns of each gene. The x axis shows the coordinates of chromosome 10

In making the case–control association study using the data from the Sigma Project (see Methods), we found several risk alleles reported before (Fig. 1). For example, the rs7903146 risk allele exhibited a high association with T2D and has been associated with Mexican and Asian populations [1, 24]. Other possible risk alleles (rs7896811, rs11196199, rs114863326, rs11196203, rs72826075 and rs35011184) were located within the cutoff limits from the Manhattan plot, and although no studies have linked these mutations with T2D, a duplication of this region (including the mutations described above) has been associated with intellectual and developmental disabilities [25]. The values of r^2^ in the intronic regions (1 to 4) of the TCF7L2 gene were less than 0.4 when we used only the African population. By contrast, we observed an increase in the values of r^2^ (Fig. 1b) in the same region when we used only the American population, and the range of values of r^2^ was 0.1 to 1. This increase in r^2^ could be related to the association with T2D. Moreover, due to the characteristics of the Sigma Project samples (ancestry controlled), the correlation between the loci associated with T2D in the intronic regions could be under selective constraints in the Amerindian populations. The TCF7L2 gene is located between two recombination hotspots (solid blue line in Fig. 1a and b). The first hotspot is upstream of the TCF7L2 gene and begins in the VTI1A and LOC103344931 genes. The second hotspot is located in exon 14 of the TCF7L2 gene. Most of the intronic variants from the Indigenous population (Fig. 1c) were located in the second recombination hotspot; however, no selective sweep peak was observed in this hotspot. The sweep peaks in the TCF7L2 gene were located in exon 5 and intronic region 5–6 (Fig. 1c). The exon 5 peak, was flanked by the r^2^ significant values and it was found in a adjacent region with XP-EHH significant values (Additional files 1, 2 and 3). These sweep peaks were previously associated with T2D and could explain the decrease in variability in the Sigma Project samples and the lack of variability in the Indigenous population in this particular region.

Across the entire region analyzed we observed several recombination hotspot peaks (solid blue line in Fig. 1c); however, we identified overlap between the selective sweep peaks (identified for several methods, Additional files 1, 2 and 3) and the recombination hotspots (elliptic dashes in Fig. 1c). The first zone of overlap was located between the upstream and downstream regions of the *ZDHHC6* gene and included two hotspots. The second zone of overlap was larger than the first and contained the *HABP2*, *NRAP*, *CASP7*, *PLEKHS1*, *MIR4483*, *DCLRE1A* and *NHLRC2* genes. This zone also included two recombination hotspots. The segment delimited by both zones had hits with a GWAS database (Fig. 1c), including associations with breast cancer, T2D and serum dimethylarginine levels. The upstream region of the *HABP2* gene is associated with migraines without aura, and the intergenic region between the *PLEKHS1* and *MIR4483* genes is associated with obesity − related traits. These associations with disease may be due to lower frequencies or the action of purifying selection to maintain the sweep patterns obtained from the recombination.

### Genomic distribution of mutations in the Indigenous population

**Fig. 2:**
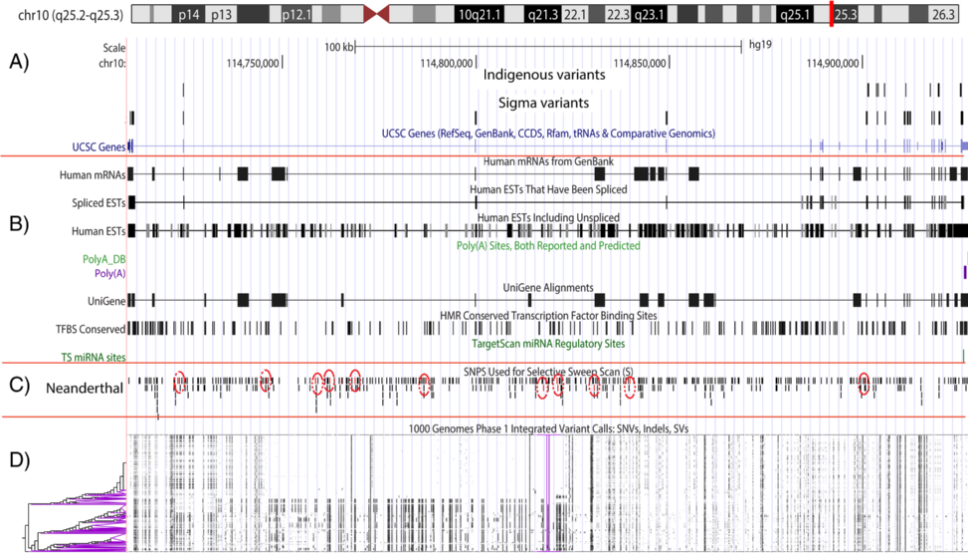
Distribution and patterns of SNPs in the TCF7L2 gene. At the top is the x axis, which comprises the coordinates of chromosome 10. **a**) Distribution of SNPs identified in the Indigenous population along the TCF7L2 gene; mutations identified in the Sigma Project and UCSC gene description, i.e., the number of exons and introns. **b**) Human mRNAs, the location of mRNA along the TCF7L2 gene; Splice ETs, chromosomal position of Human ETs with splice; Human ETs, distribution of human Ets; Poly(A) sites, dispersion of sites associated along a tissue-specific region; UniGene, alignments of all mRNA and EST tracks from TCF7L2 transcription; TFBS (Transcription Factor Binding Sites) conserved, in the figure, each box represents one site conserved in the human/mouse/rat alignment; TargetScan miRNA Regulatory Sites, this track shows conserved mammalian microRNA regulatory target sites in the 3’ UTR regions of TCF7L2. **c**) Genomic location of variants used in the identification of selective sweeps in the Neanderthal genome. Dashed red circles indicate mutations that are under selective sweeps. **d**) Genetic differentiation and patterns of variation in the TCF7L2 gene identified in the 1000 Genomes Project for all populations

Purifying selection acts not only in the coding regions of the TCF7L2 gene but also in the introns. As a result of selection, we observed only 1,137 variants along two megabases of chromosome 10 (including TCF7L2) of the Sigma Project exomes (data not shown). We observed a decrease in the variability of the TCF7L2 gene (80 variants) in the Indigenous population. The localization of SNPs along TCF7L2 gene was not constant, i.e., regions with low and high density of mutations were observed. The last exons (6–14) of the gene harbored the higher density of SNPs (Fig. 2a). We observed the same distribution of SNPs in the Indigenous population as in the Sigma samples; most of the variants were located in intronic areas. Therefore, we searched the intronic SNPs in RNA databases (see Methods). We did not observe hits of the intronic variants against human mRNAs, despite the higher density poly(A)-sequencing in the last exon of the TCF7L2 gene (Fig. 2b), where the variation in the Indigenous population was concentrated. The same result was obtained when we searched intron variants in the regulatory sites database at http://mirdsnp.ccr.buffalo.edu/browse-genes.php. However, more than one causal variant associated with T2D was located in introns 4–6; for example, the rs7903146 risk allele (described above) had an effect on enhancer activity, resulting in higher TCF7L2 gene expression [26]. Comparing the sequence of the TCF7L2 gene against the Neanderthal genome (http://neandertal.ensemblgenomes.org/Homo_sapiens/Location/Genome) (Fig. 2c) revealed a random distribution of variants. The intronic region (4 to 6) provided an agglomeration of selected mutations (selection scanning). This agglomeration may suggest selection before the Neanderthal-human speciation. At the human population level, the intronic region of 4 to 6 exhibited greater differentiation in SNP patterns (bottom of Fig. 2d). This differentiation could be the product of the action of selective sweeps in fixing low-frequency mutations associated with T2D.

### Haplotype patterns and phylogenetic networks

**Fig. 3:**
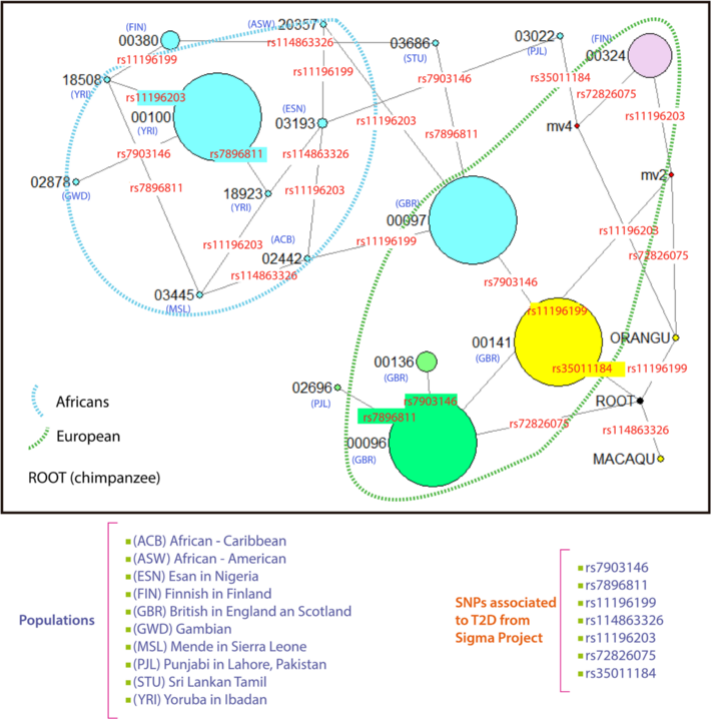
Network median joining. Haplotype network based on the sequences of variants (rs7903146, rs7896811, rs11196199, rs114863326, rs11196203, rs72826075 and rs35011184) of the samples of the 1000 Genomes Project associated with T2D. The nodes are the haplotype profiles and mv-nodes are the missing haplotypes. The outgroup included nucleotides from chimpanzee, root, macaque and orangutan. Each color indicates a possible haplotype profile; the color was randomly assigned. The size of the sphere is related to the frequency of the haplotype patterns. The dotted blue line includes the haplotypes from the African population, and the dotted green line contains the haplotype patterns of the European population. The bottom left of the figure shows the populations included in the network. The bottom right lists the mutations associated with T2D from the Sigma Project.

Allelic frequencies are subject to selective and recombination processes. For example, the diversity pattern of the rs7903146 risk allele suggests a positive selective sweep that may have rapidly driven fixation in East Asians and decreased the frequencies in Europeans and Africans [12]. We observed an extension of the positive selective sweep near the rs7903146 SNP comprising the intronic region 4 to 6, and a possible effect of this extension would be a decrease in the frequencies of the SNPs associated with T2D (rs7896811, rs11196199, rs114863326, rs11196203, rs72826075 and rs35011184). To determinate the relationships among the different haploid genotypes associated with T2D, we performed a network haplotype (see Methods). For this analysis, we included the genotypes of macaque, orangutan and chimpanzee, with the latter as the outgroup (Root). Although we included a root (Outgroup) on network (Fig. 3), all internal nodes exhibited reticulations, hindering the interpretation of the hypothetical ancestors. The internal nodes that surrounded the root were grouped (green dashes) by their haplotype profiles. The European haplotype pattern presented major frequencies, including at the rs7903146 SNP. Other groups (Fig. 3, blue dashes) were formed from haplotype 00100, producing nodes with lower frequencies in the African populations. The rs7903146 risk allele was located in all networks, suggesting that it is an ancestral haplotype and possibly fixed in all populations; however, this risk allele was not derived from the root (chimpanzee). By contrast, the possible rs114863326 risk allele was observed between the nodes from the chimpanzee and macaque and was located only in the African population group (blue dashes). The predominance of rs114863326 within the African group would suggest a possible selective sweep to each population mediated by intra-species recombination.

**Fig. 4:**
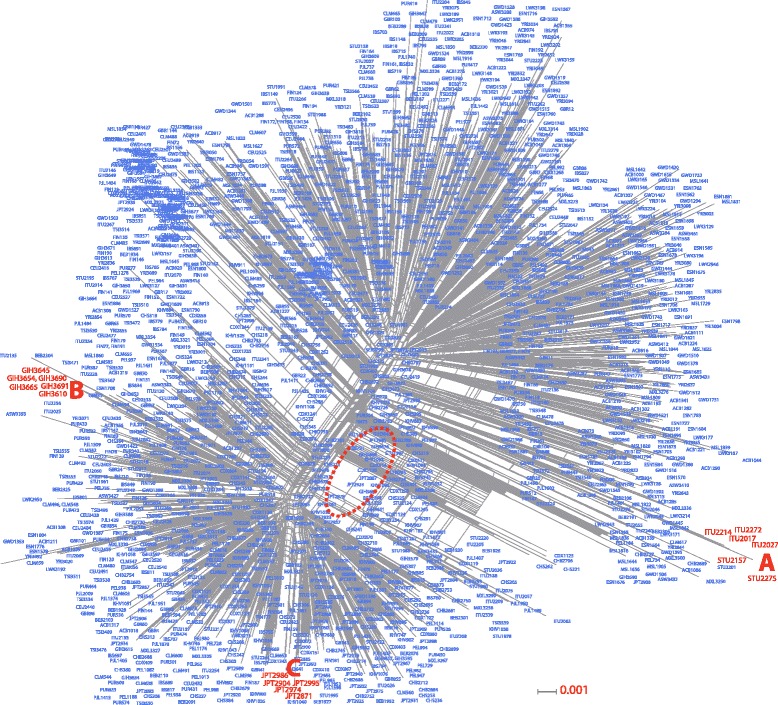
NeighborNet. Network analysis was based on 6061 mutations in the TCF7L2 gene identified from the samples of the 1000 Genomes Project. The dashed red circles indicate the reticulations between the nodes. The letters **a**, **b** and **c** are clusters with phylogenetic coherence. The scale is located at the bottom of the network and shows their substitutions by site

The reticulations on the internal nodes are produced by recombination effects. The rapid growth of the human population also facilitated the spread of rare variants among different races. The presence of recombination hotspots inside and outside the TCF7L2 gene suggests constant genetic flow, and the phylogenetic interpretation is consequently unclear. We therefore constructed a NeighborNet tree (see Methods) based on the variation obtained from the 1000 Genomes Project. As shown in Fig. 4, two principal groups were formed, each including several types of human populations. This phylogenetic incoherence was derived from the reticulations (dashes red) within and between both groups. Despite the reticulations, small groups maintained coherence (Fig. 4), for example, the Indian population with cluster A (ITU, Karimnagar) and B (GIH-ITU, Gandhinagar and Karimnagar). Cluster C included a Japanese population (JPT). These clusters with phylogenetic coherence suggest that the majority of the observed recombination events are ancient.

## Discussion

TCF7L2 harbors variants with the strongest effect on T2D. The phenotypic changes associated with the risk genotype suggest that T2D arises as a consequence of impairment of several vital functions in the pancreatic islet [27]. In addition, the synthesis and processing of proinsulin and possibly the clearance of proinsulin and insulin are regulated by TCF7L2 [28]. Due to the functional importance of TCF7L2, any mutation in either the coding or noncoding regions could change its function. Recently, the common noncoding variant rs7903146 (risk T-allele) was associated with increased TCF7L2 expression and decreased insulin content and secretion [28]. This variant has been associated with T2D in several ethnic groups, such as Japanese, Indian, Tunisian, European and Mexican [1, 24, 29–31]. By replicating the case–control association analysis from the Sigma Project, the rs7903146 risk allele was also associated with T2D, and other possible risk alleles placed on introns 4–6 (rs7896811, rs11196199, rs114863326, rs11196203, rs72826075 and rs35011184) were located in the limits of the cutoff values of the Manhattan plot. The presence of the rs7903146 SNP in several populations may suggest an ancestral character; however, the association of this variant with T2D in populations with controlled ancestry might suggest an increased rate of recombination in a specific population. The dispersion of the possible risk allele rs35011184 in a given population may be mediated through recombination or by the occurrence of new mutations within the region [32, 33]. Although LD has been reported in the TCF7L2 gene, rs7903146 exhibited moderate LD in the European populations (r^2^= 0.73) [34]. By contrast, we observed higher LD of the 4–6 intronic region (including rs7903146) associated with T2D (0.5 to 1) in the Latino population (r^2^= 0.5 to 1) than the African population (r^2^= 0.2 to 0.5), and the SNPs in LD were not associated with T2D, suggesting a vertical inheritance of genotypes.

Phylogenetic analysis of exon 4 suggested that the rs7903146 risk allele is an ancestral character and tends to recombine with a variety of haplotype backgrounds, particularly in the relatively diverse West African population [12]; however, the analyses of Network median joining of the intronic variants indicated an increase in the recombination of rs7903146 because it was mainly placed in two of the groups. Moreover, the possible risk allele rs114863326 also exhibited ancestral character and was located next to the root taxon (chimpanzee). Notably, this variant was only localized in a cluster of the African population, suggesting that this variant tends to recombine with this group. The increase in reticulations in the haplotype network in the intronic regions could indicate a decrease in selection forces compared to the coding regions; however, we did not observe differences in the reticulations of the neighborNet tree from 6,061 variants (in the TCF7L2 gene) in mostly exonic regions obtained from the 1000 Genomes Project. The only phylogenetic consistency was observed in small groups belonging to the same population. The excess of phylogenetic inconsistency across the TCF7L2 gene could be the result of recombination hotspots in the surrounding region (1 Mb) of the TCF7L2 gene. In particular, two recombination peaks flank the gene at the 5’ and 3’ ends. Some apparent recombination hotspots could be an artifact of variable population size over time (demographic effects) because these hotspots were identified assuming a particular model of human history, and one of the assumptions of that model is that the effective population size of humans has remained constant throughout history [35]. The inferred recombinant hotspots in gene regions that also appear to have undergone positive selection [36] may not be due to non-uniform densities of meiotic recombination but may simply be a by-product of positive selection. Therefore, selective sweeps do make a significant contribution to the patterns of LD in the human genome [37].

Selective sweeps can increase the genetic differentiation among populations, cause allele frequency spectra to depart from the expectation under neutrality, and also alter the allele frequencies of single nucleotide polymorphisms (SNPs) in the vicinity of the selected allele [38]. We observed two peaks of selective sweeps in exon 5 and intron region 5–6 of the TCF7L2 gene in samples from the Sigma Project with Amerindian ancestry greater than 70 %. This peak is within intron region 4–6 (associated to T2D), which covers approximately 81 % (178,063 bp) of the TCF7L2 gene. Because more than half of the gene is constantly under sweep selection, the reduction or elimination of variation among the nucleotides in neighboring regions could explain why most of the mutations (40 variants) observed in the Amerindian population occurred in intron 7 to 15 and why only six mutations were observed in introns 4 and 6. Forty of the mutations were not observed in the 1000 Genomes Project or the Sigma Project, and all had a low frequency (rare variants). Moreover, four of the variants were identified in the 1000 Genomes Project, and the rs176632 (T/C) and rs56913138 (G/T) SNPs exhibited a MAF of 0.99 and 0.19, respectively. In the Sigma Project, the two mutations (rs77961654 C/A and rs61724286 C/G) in exon 15 exhibited the lowest MAF of 0.08 and 0.0009, respectively. All mutations in the Amerindian population identified in both databases (1000 Genomes Project and Sigma Project) are of ancestral character and survived genetic drift, possibly by strong positive selection. Most of the peaks of selective sweeps were identified downstream of the TCF7L2 gene, including the following genes: HAPB2, NRAP, CASP7, PLEKHS1, MIR4483, DCLRE1A and NHRLC2. This region was associated with diseases from the GWAS catalog such as obesity-related traits and Gaucher disease severity. In addition, the peaks of selective sweeps overlapping with recombination hotspots could influence the selection of the TCF7L2 gene.

The impact of the TCF7L2 gene on metabolic diseases is fundamental because this gene has multiple targets in the pathways for insulin synthesis [28]. The hepatic expression of TCF7L2 is decreased in diabetic patients as their body mass index (BMI) increases [39]. Moreover, studies in mice have revealed that the regulation of TCF7L2 expression varies in different tissues. In mice fed a high fat diet (HFD), the expression levels of TCF7L2 were upregulated in pancreatic islets but down-regulated in the liver [40]. Furthermore, the TCF7L2 gene displays a complex pattern of spliced variants, with several alternative exons and splice sites, resulting in splice variants that are distributed and expressed in a tissue-specific manner [41–44]. The presence of the rs61724286 (C/G) splice variant identified in Zapotecan and mixed race (group with less Amerindian ancestry) populations in conjunction with the rs77961654 (C/A) missense variant (present in three indigenous populations) may not be tolerable for function of the TCF7L2 gene. The function of this gene is also impacted by the high-fat, caloric-dense diets and sedentary lifestyles common in our society. Although most mutations surviving the processes of selective sweeps in the Indigenous populations were intronic and had no immediate functional impact, the accumulation of mutations, particularly in exon 15, could have a damaging effect on the regulation of metabolism by the TCF7L2 gene.

## Conclusions

The TCF7L2 gene is the locus most strongly associated with T2D risk in different populations [26]. In addition, evidence of LD and mutations in the TCF7L2 gene [34] that are under selective processes exist [12]; however, ancestry has not been controlled in most studies, and evolutionary processes in Amerindian populations have not been evaluated. In this work, we identified and analyzed the nucleotide variants in three indigenous populations: Mazatecan, Nahua and Zapotecan. Although the number of sampled individuals in each of the three groups was low (30–60) and varied, the high Amerindian ancestry of the indigenous population allowed us to detect changes in the allelic frequencies in the Mexican population (Sigma Project) and the rest of the human population (1000  Genomes Project). Thus, we observed an asymmetric distribution of nucleotide variants in the 5’ and 3’ ends of the TCF7L2 gene. Most of the mutations were located within intron 6–7 onwards. The only two coding mutations were located in exon 15. The lack of diversity in the Indigenous populations in intronic regions 1–6 could be due to a selective sweep because we identified two selection peaks in the samples from a Mexican Mestizo population (Sigma Project) with Amerindian ancestry higher than 70 %. The first peak was located in exon 5, and the second was located in intron 5–6. Additionally, the two selective peaks were placed within the area (intronic 4–6) containing mutations associated with T2D, including the rs7903146 risk allele, which was the most frequent allele not only in the samples from the Sigma Project but also in the various human populations. The reticulations identified in the network analysis, either in the variants associated with T2D or all of the variants of the TCF7L2 gene in the samples of the 1000 Genomes Project, suggest that the dispersion of rs7903146 in human populations is due to recombination. This hypothesis is supported by the recombination hotspots located in the upstream region and 3’ end of the TCF7L2 gene. Furthermore, the overlap of the sweep peaks with the recombination hotspots in the area downstream of the gene, specifically in an area associated with obesity-related traits and Gaucher disease severity, indicates that the TCF7L2 gene is part of a strongly selected region in which rare variants that survive selective sweep could be genetic sources that modify metabolism.

## Methods

### Subjects

The Amerindian population, based on self-reporting, comprised 60 Mazatecan from San Lorenzo Cuaunecuiltitla, Oaxaca (Mexico), 34 Nahuas from Xoteapa, Veracruz (Mexico) and 30 Zapotecan from the Mexican Diversity Project. Henceforth, these populations are referred to as the Indigenous population. Another group of 30 mixed-race Mexicans (Mestizo population) from the Mexican Diversity Project [45] participated. All individuals gave informed consent before they were included in the study. Mestizo and Zapotecan participants gave written consent. Verbal consent from Nahua population was taken and written consent from Mazatecan population was taken. Mestizos and Zapotecan samples were taken with anonymazed consent; All the individuals were 18 years or older. The age range was: Nahua from 37 to 81 years old, Mazatecan from 22 to 78 years old.

### Sequencing

DNA was extracted using the QIAamp DNA Blood Maxi Kit, QIAGEN. We designed primers for each exon that also covered part of the introns surrounding the exon (Additional file 4). Sequencing was performed using the Sanger method on an Applied Biosystems sequencer (3730xl DNA Analyzer) with Big dye terminator (standard method).

### Databases

The variability (SNPs) of chromosome 10 was obtained from the 1000 Genomes Project ((v4.20130502) ftp://ftp.1000genomes.ebi.ac.uk/vol1/ftp/release/20130502/). This project contains five super-populations: African (AFR), Ad Mixed American (AMR), East Asian (ASN), European (EUR), and South Asian (SAN). The numbers of sampled individuals were 661, 347, 504, 503 and 489, respectively. The family relationship is included in the *.bed files along with the gender and geographic origin of the individuals.

To assess variability inside the Latino population, particularly on chromosome 10, we used the Sigma Project database [1], which includes 4,060 whole-exome sequences of individuals. This database consists of 1954 controls, 1905 T2D cases and 201 individuals without ascertainable clinical histories. The Sigma Project comprises four databases: DMS (Diabetes in Mexico Study), MCDS (Mexico City Diabetes Study), MEC (Multiethnic Cohort) and UIDS (UNAM/INCMNSZ Diabetes Study). We used the UIDS database because of the protocols used to identify individuals with T2D. Cases were recruited at the outpatient diabetes clinic of the Department of Endocrinology and Metabolism of the Instituto Nacional de Ciencias Médicas y Nutrición Salvador Zubirán (INCMNSZ). An important feature of this database is that the Amerindian ancestry of the individuals was higher than 70 % (ancestry was calculated previously by exome-chip sequencing). A high Amerindian ancestry eliminates the influence of other populations in the genetic analysis. The analyzed sequence included only the coding regions (exome-sequencing) and also spanned the intronic regions adjacent to exons.

### Determination of SNPs

The quality of Sanger sequencing (q-value) of the Indigenous populations was assessed by the fastqc program [46], with pretreatment of the reads using in-house Python scripts. The sequencing chromatograms were analyzed by the PolyPhred program [47]. We used a minimum q-value of 20 and the following parameters: −f 16, −score 90, −window 15. The output PolyPhred was processed by perl scripts [48] for mutation screening. The dbSNPs database v. 138 [49] was used to annotate the SNPs identified by the perl scripts.

### Association of case–control

Replication of the association test of case–control for T2D in Latinos with Amerindian ancestry greater than 70 % (described above) was performed by the plink program [50] with the parameters provided in the Sigma Project [1].

### Genomic region for evolutionary analysis

The area of study for selective sweep analysis and recombination focused only on chromosome 10 because this region harbors the TCF7L2 gene. The region was defined by referring to the human genome (hg19 version) and delimited with the coordinates: 113,925,369 – 115,925,369. This region encompasses the following genes: GPAM, TECTB, MIR6715A, MIR6715B, GUCY2GP, ACSL5, ZDHHC6, VTI1A, MIR4295, LOC103344931, TCF7L2, HABP2, NRAP, CASP7, PLEKHS1, MIR4483, DCLRE1A, NHLRC2, ADRB1 and CCDC186.

#### Identification of selective sweeps

Based on the variation identified in chromosome 10 from the whole-exome-sequencing of the Sigma Project (described above), we constructed a nucleotide sequence composed mainly of variation (SNPs) for each individual. If a mutation was not observed at a particular position for an individual in the alignment of all samples, we used the existing nucleotide in the hg19 reference genome (The Genome Sequencing Consortium). This process comprised two parts. First, we extracted the nucleotide variation for each individual using the pseq program [50] with flags v-view --geno –mask. Next, the nucleotides were concatenated through perl scripts without losing the order of chromosome 10. In the second part, we revised and formatted the alignment to analyze selective sweeps using perl scripts. Selective sweeps were identified by traditional and whole-genome tools.

The traditional tests used: iHS [51], XPEHH [51] and nSL [52]. The iHS scores were calculated and normalized using selscan program [53] with parameters: −-max-extend 1000000 (maximum EHH extension in bp), −-max-gap 300000, −-gap-scale 30000, −-cutoff 0.05 (EHH decay cutoff) and --alt. We used the recombination map of Altshuler [54]. The output results for each SNP were then frequency-normalized over chromosome 10 using the script norm, provided with Selscan. This normalization was also done using default parameters: −-bins 100 (number of frequency bins). The fractions of SNPs with |iHS| values above 2.0 were retained.

The nSL scans were performed and normalized using Selscan program [53] following the same procedures used for iHS. The iHS statistic compares the integrated EHH (Extended Haplotype Homozygosity) profiles between two alleles at a given SNP in the same population. It is noteworthy that the nSL test uses a slightly different approach to screen for EHH than the original iHS test and is robust to variation in recombination frequency, also identify selective peaks under a number of different selection scenarios, most notably in the cases of sweeps from standing variation and incomplete sweeps [52].

The XP-EHH (Cross Population Extended Haplotype Homozygosity) statistic compares the integrated EHH profiles between two populations at the same SNP [55] and is expected to be more reliable if a reference population with a similar demographic history is available, and if the allele under selection is close to fixation in one of the populations. XP-EHH score were calculated by Selscan program [53] with parameters: −-xpehh –max-gap 300000 –gap-scale 30000 and --alt. We compared Latino population vs African population reference of the 1000 Genomes Project. The scores for each SNP were then frequency-normalized over chromosome 10 using the script norm, provided with Selscan. We fit the XPEHH scores distribution using General model Gauss with one component by Matlab program [56]. Since that the negative XPEHH suggest selection happened in reference population, we identified the two Gauss distribution tails with 95 % confidence. SNPs passing positive threshold are candidates for selection in Latino population and those passing negative threshold are in African population.

The whole-genome tools: The ω statistic proposed by Kim and Nielsen [21] was able to accurately localize the selective sweeps by a likelihood (ML) framework that uses linkage-disequilibrium (LD) information. OmegaPlus program [57] has a high-performance implementation of the ω statistic at genome level and was utilized for scan to chromosome level the selective sweeps, with parameters -grid 47370, −minwin 1, −maxwin 5, −threads 20, −impute N, −seed 20, and -all. The peaks of selective sweeps from OmegaPlus were those that exceeded the upper confidence interval calculated by the function dfittool from the Matlab program [56].

### Calculation of linkage disequilibrium (LD) and integration of genetics analysis

LD was calculated for chromosome 10, particularly for the regions of the TCF7L2 gene. We extended this region 1000 kb downstream and upstream of the TCF7L2 gene (described above). The plink program [50] was used with default parameters; however, to rule out the influence of non-Latino populations, we calculated LD based on the African population of the 1000 Genomes Project (because it is the oldest population) and another analysis with the Latino population only.

The mutations from the Sigma Project localized to the extended region (described above) and were searched against a GWAS database to identify the diseases associated with this genomic region. Additionally, the peaks of selective sweeps (determined above) and mutations of the Indigenous population previously identified were integrated by the LocusZoom program [58].

### Haplotype network and phylogenetic network analysis

To determine the relationships among the different genotypes associated with T2D (describe above), we performed Network median joining using the Network program [59]. We used the following modules: Start contraction, Median joining and MP calculation. For the Outgroup, we included orangutan (GCA_000001545.1), chimpanzee (GCA_000001515.4) and macaque (MMUL 1.0) sequences. Mutations in the TCF7L2 gene (6,061 mutations) from the 1000 Genomes Project were concatenated, and a fasta file was constructed. Next, a neighborNet tree was constructed by the Splistree program [60] using default parameters.

### Functional impact of mutations

The mutations were analyzed based on their genomic coordinates in the ANNOVAR [61] and SNPSIFT [62] databases. Both databases include several algorithms (nine in all) that predict the functional effect. We selected a mutation if it was positive in at least 4 algorithms. The filters were numbers or letters as follows: POLYPHEN2 (“P and D”), LRT (“D” and “ < 0.0001”), MutationTaster (“A and D”), MutationAssessor (“high, medium and low”), FATHM (“ < −1.5”), GERP++ (“ > = 5”), SIFT (“ < 0.05”), phylop (“ > = 3”) and PROVEAN (“ < −2.5 Deleterious probably” and “ < −4.1 Deleterious”).

### Effects of mutations on gene expression

The SNPs could create, destroy, or modify the efficiency of miRNA binding to the 3’UTR of a gene, resulting in gene dysregulation. Consequently, we explored each mutation identified in the Indigenous population in the following databases: miRdSNP [63], mrSNP [64] and RNAsnp [65]. We plotted our results using the UCSC browser (http://genome.ucsc.edu/cgi-bin/hgGateway).

### Ethics approval and consent to participate

This study was approved by the Research Committee, Research Ethics Commitee and Institutional Biosafety Commitee from the National Institute of Genomic Medicine. All individuals gave informed consent before they were included in the study. Mestizo and Zapotecan participants gave written consent; Mestizos and Zapotecan samples were taken with anonymized consent; Verbal consent from Nahuatl population was taken and written consent from Mazatecan population was taken. The consent from Nahuatl population was verbal, because the samplings were made with the consent of the leaders of the ethnic group and each participant gave his approval to participate in the study. The oral approval was obtained with a translator and answers were given to all questions about the investigation.

## Additional files

Additional file 1: Details of selection analysis by several methods. (DOCX 14.8 kb) Additional file 2: Analysis of Selection Sweeps (iHS, nSL, w and XP-EHH). (JPG 421 kb) Additional file 3: GWAS Catalog SNPs in Region. (DOC 61.5 kb) Additional file 4: Primer used to sequencing of TCF7L2 gene exons. (TXT 3.24 kb)

## Abbreviations

GWAS: genome-wide association study
HET: heterozygosity
MAF: minor allele frequency
REF/VAR: reference allele/mutated allele
REFMIN: minimum frequency to the reference
SNP: single nucleotide polymorphism
T2D: type 2 diabetes mellitus
