# Supplementary material for: Rare intronic variants of TCF7L2 arising by selective sweeps in an indigenous population from Mexico
Source: BMC Genet. 2016 May 26;17:68. doi: 10.1186/s12863-016-0372-7 (PMC4880969; doi:10.1186/s12863-016-0372-7)
Supplement: Additional file 1: — Details of selection analysis by several methods. (DOCX 14.8 kb) [file 12863_2016_372_MOESM1_ESM.docx]

**Details of selection analysis by several methods.**

For XP-EHH, we used the Africans population as reference and the Latino population as target (Mentioned in the main text). Since the negative scores of XP-EHH means that selection events occurred in the ancestral population, we used General model Gauss with one component to fit the distribution of normalized XP-EHH score. Using this fit, we obtained the cutoff of XPEHH > 1.5 and XPEHH < -0.085 at 95% chromosome-wide significance levels.


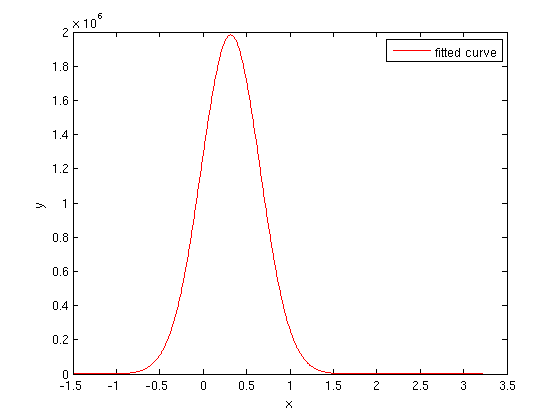

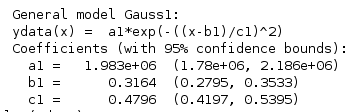


Figure1. Fitted distribution of normalized XP-EHH scores (red). The Gaussian function used is P(x) = a1 * exp (-((x-b1)/c1)^2), the values for the parameters are given in the legend.

Next figure (Additional file 1b), we showed only significant scores of all tests to identify selective sweeps. The selection sweeps block consisted of the statistical iHS, nSL, w and XP-EHH. The positive score of XP-EHH were located in the regions adjacent to the TCF7L2 gene, identified by yellow blocks. The clustering of statisticians to identify selective sweeps in a region of the chromosome, might suggest a greater effect of selection and in figure were denoted with dark yellow. Otherwise, few selective peaks clustered means soft selective sweeps (light yellow block). TCF7L2 gene showed some peaks of selection sweep in a region with r^2^ significant values, which were obtained only from Latino population. Finally, peaks of recombination rates were plotting if they were higher to 10.
