# Supplementary figures and images for: Rare intronic variants of TCF7L2 arising by selective sweeps in an indigenous population from Mexico
Source: BMC Genet. 2016 May 26;17:68. doi: 10.1186/s12863-016-0372-7 (PMC4880969; doi:10.1186/s12863-016-0372-7)

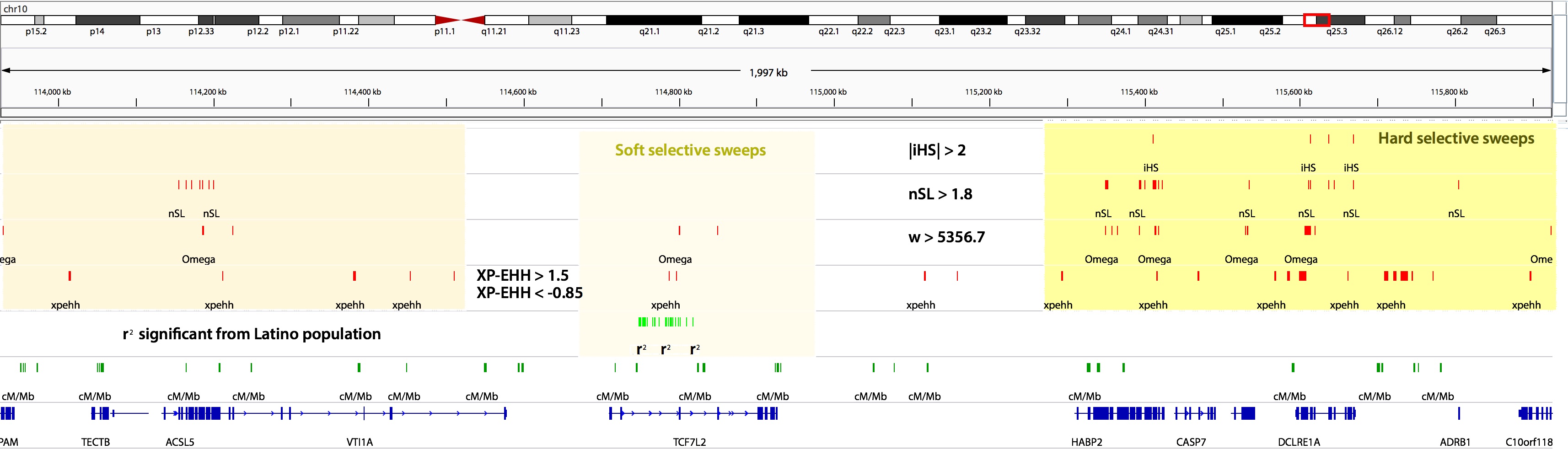

Supplement: Additional file 2: — Analysis of Selection Sweeps (iHS, nSL, w and XP-EHH). (JPG 421 kb) [file 12863_2016_372_MOESM2_ESM.jpg]
