# Supplementary material for: Rare intronic variants of TCF7L2 arising by selective sweeps in an indigenous population from Mexico
Source: BMC Genet. 2016 May 26;17:68. doi: 10.1186/s12863-016-0372-7 (PMC4880969; doi:10.1186/s12863-016-0372-7)
Supplement: Additional file 3: — GWAS Catalog SNPs in Region. (DOC 61.5 kb) [file 12863_2016_372_MOESM3_ESM.doc]

GWAS Catalog SNPs in Region
